# Genome characterization of the *Microbacterium foliorum* bacteriophage CookieDog (cluster GA) isolated in Florida, USA

**DOI:** 10.1128/mra.01241-25

**Published:** 2026-05-12

**Authors:** Luke Kolinsky, Paige Manuel, Angeleudys Miranda, Johanna Beason, Ernie Arteaga, Jose Pardo, Lara Becerra-Reynundo, Patricia Waikel, Jaime Mayoral

**Affiliations:** 1 Department of Biological Sciences, Florida International University 5450, Miami, Florida, USA; Queens College Department of Biology, Queens, New York, USA

**Keywords:** bacteriophages, genome announcement, siphovirus, microbacterium

## Abstract

The *Microbacterium* bacteriophage CookieDog was isolated from a soil sample in Miami, FL. CookieDog has a genome length of 38,761 bp, contains 66 putative genes, and is assigned to actinobacteriophage cluster GA based on shared gene content. Transmission electron microscopy shows a siphovirus morphology.

## ANNOUNCEMENT

*Microbacterium foliorum* NRRL B-24224 is a host for the discovery of new bacteriophages (1) to explore the diversity and ecology of actinobacteriophages, including phage-encoded functions. The 678 phages in phagesdb.org isolated and sequenced on *M. foliorum* are grouped in 18 clusters and 7 singletons. A new phage, CookieDog, was discovered in Miami, FL, using *M. foliorum*.

**Fig 1:**
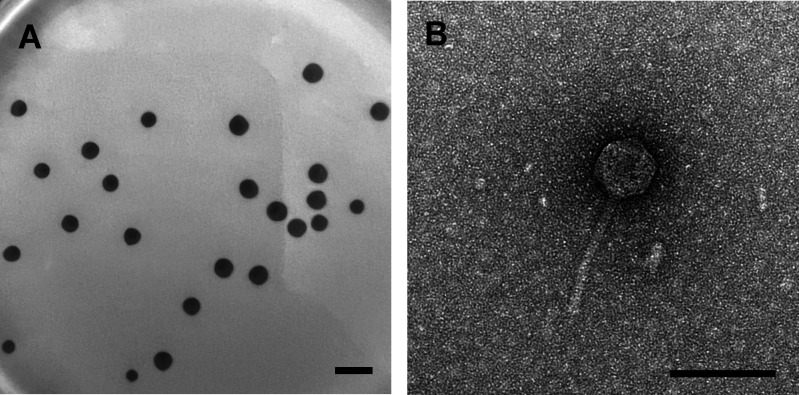
Plaque morphology of bacteriophage CookieDog and transmission electron microscopy. (**A**) Plaque assay showing clear and well-defined plaques after incubation at 30°C for 24 h, scale bar = 5 mm. (**B**) Transmission electron microscopy of CookieDog, scale bar = 100 nm. Phage samples were negative-stained by placing them onto 200-mesh formvar-covered, carbon-coated copper grids (EMS, Hatfield, PA, USA), incubated for 1 min, rinsed with ultrapure water, and then stained with 2% uranyl acetate. Images were taken at 100 kV using a Hitachi HT7800 120 kV TEM with an AMT NanoSprint15B digital camera.

CookieDog was isolated from a soil sample collected at a marsh near Homestead, FL (GPS coordinates 25.39746 N, 80.57309 W). The sample (~15 g) was resuspended in 20 mL of peptone-yeast extract-calcium (PYCa) liquid medium, incubated at 30°C for 2 h, and filtered through a 0.22 μm filter. Phages were enriched by inoculating the filtrate with *M. foliorum* and incubating at 30°C for 48 h. The enriched sample was filtered, and the filtrate was plated in PYCa top agar with *M. foliorum*. Individual plaques were picked and purified through two rounds of plating. CookieDog plaques were 2.39 ± 0.48 mm (*n* = 10) in diameter with defined circular margins (Fig. 1). Transmission electron microscopy showed a siphovirus morphology (Fig. 1), with a capsid diameter of 57.67 ± 0.58 nm (*n* = 3) and a tail length of 117.83 ± 0.29 nm (*n* = 3).

DNA was extracted from the phage lysate using the Quick-DNA Viral Kit (Zymo), prepared for sequencing using the NEB Ultra II FS Kit, and sequenced using Illumina NextSeq 1000 (XLEAP-P1 kit), yielding 4,075,973 100-base single-end reads, which constituted a 10,325-fold coverage. Raw reads were trimmed with Cutadapt 4.7 (using the option –nextseq-trim 30) and filtered with skewer 0.2.2 (using the options -q 20 -Q 30 -n -l 50) prior to assembly, and the genome end was determined as previously reported (2). Reads were assembled using Unicycler 0.5.0, and genomes were checked for completeness using Consed (3, 4).

CookieDog’s genome was annotated using DNA Master. Gene starts were evaluated using GeneMark v3.25 (5), Glimmer v3.2 (6), Starterator v600 (http://phages.wustl.edu/starterator), and PECAAN v20241104 (https://discover.kbrinsgd.org/). Gene functions were predicted using Phamerator v.594 (7), BLASTp (8) (actinobacteriophage proteins and non-redundant sequence [nr] databases), and HHPRED (9) (PDB_mmCIF70, Pfam-A, NCBI_Conserved_Domains, and UniProt-SwissProt-viral70 databases). tRNAs were predicted using Aragorn v2.41 (10) and tRNAscan-SE v2.0 (11).

CookieDog’s genome is 38,761 bp long, has a GC content of 68.0%, and encodes 66 predicted genes. All genes are transcribed in the same direction, with the exception of a tRNA (Gly) between 30,428 and 30,502 bp, and the genome has a circularly permuted end. Based on gene content similarity, CookieDog is assigned to cluster GA (12, 13). Similar to other GA cluster phages, the first third of the genome primarily encodes predicted structure and assembly genes, while the remainder primarily contains genes predicted to be involved in replication and recombination (8). A −1 bp translational frameshift was identified at 11,135 bp within the putative tail assembly chaperone. No immunity repressor or integrase was identified, suggesting it is unlikely to establish lysogeny.

## Data Availability

CookieDog is available at GenBank (accession no. PV876955) and Sequence Read Archive (no. SRR33237334).
